# Supplementary material for: Comparison of the Biochemical Properties and Roles in the Xyloglucan-Rich Biomass Degradation of a GH74 Xyloglucanase and Its CBM-Deleted Variant from Thielavia terrestris
Source: Int J Mol Sci. 2022 May 9;23(9):5276. doi: 10.3390/ijms23095276 (PMC9103125; doi:10.3390/ijms23095276)
Supplement: Supplementary file 1 [file ijms-23-05276-s001.zip › ijms-1717688-supplementary.pdf]

Supplement

Comparison of Biochemical Properties and Roles in Xyloglucan-rich Biomass Degradation of a GH74 Xyloglucanase and its CBM-deleted Variant from *Thielavia terrestris*

Beibei Wang, Kaixiang Chen, Peiyu Zhang, Liangkun Long and Shaojun Ding *

Jiangsu Co-Innovation Center of Efficient Processing and Utilization of Forest Resources, College of Chemical Engineering, Nanjing Forestry University, Nanjing 210037, China; jason22@njfu.edu.cn (B.W.); chenkaixiang@njfu.edu.cn (K.C.); zhangpeiyu@njfu.edu.cn (P.Z.); longlk602@njfu.edu.cn (L.L.)

* Correspondence: dshaojun@njfu.edu.cn or dshaojun@hotmail.com; Tel.: +86 25 85427939; Fax: +86 25 85418873

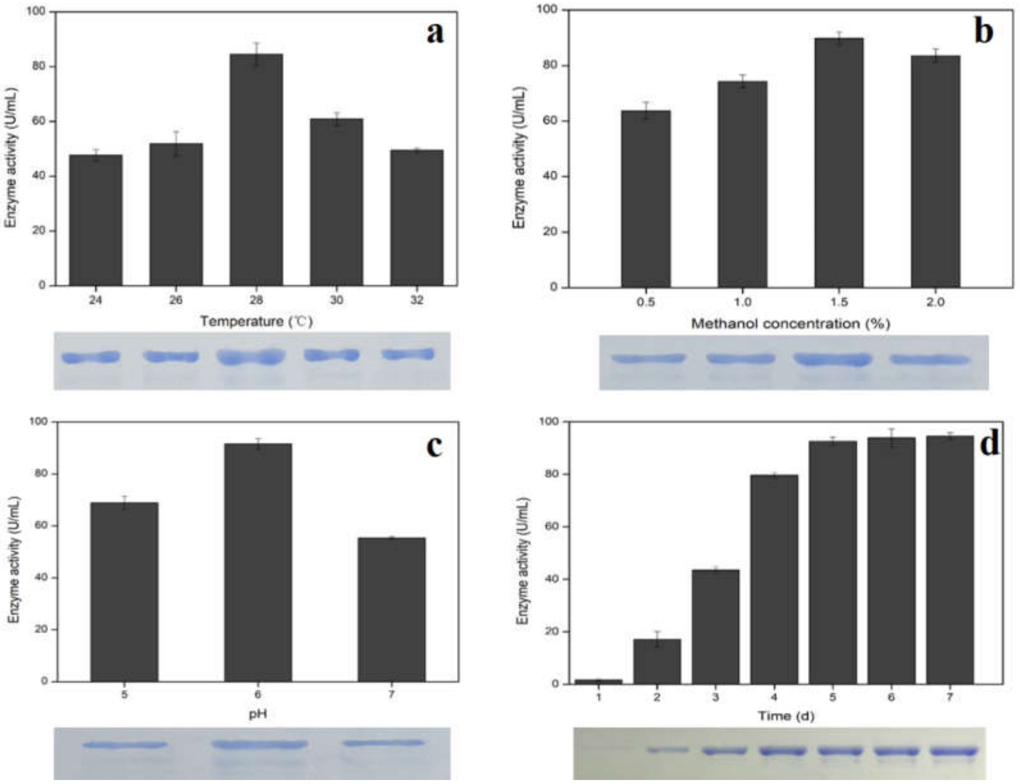

Figure S1. Effect of temperature (**a**), methanol concentration (**b**), pH of the medium (**c**) and induction time (**d**) on enzyme activity of *Tt*GH74 and expression levels shown on the gels.

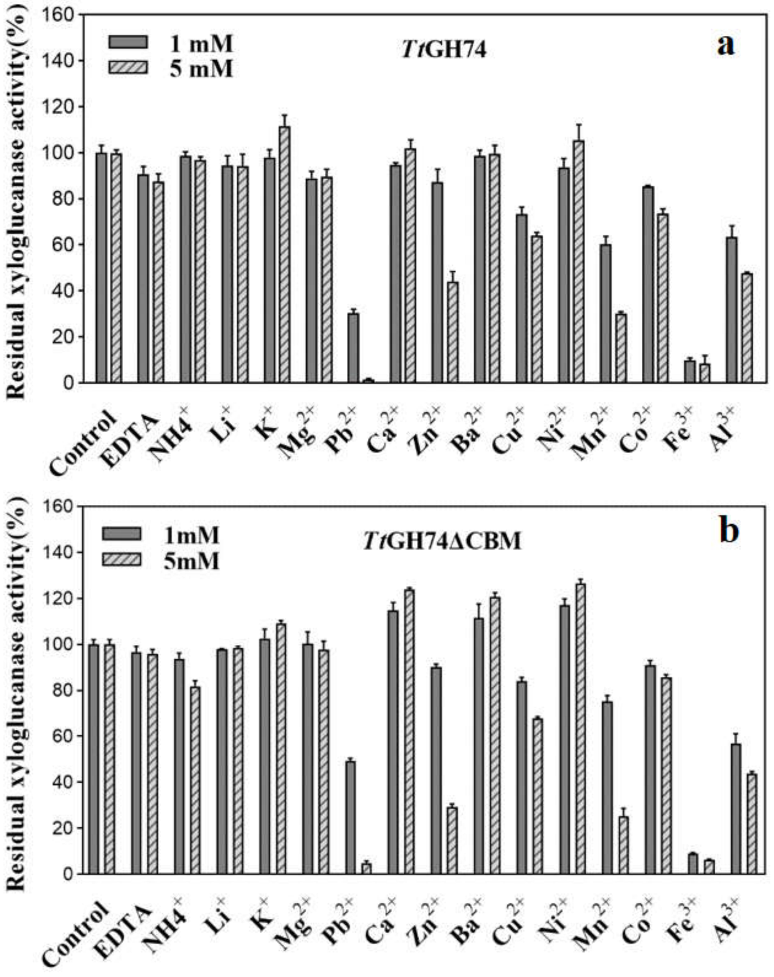

Figure S2. Effect of metals ions (1 and 5 mM) on the *Tt*GH74 (**a**) and *Tt*GH74ΔCBM (**b**) activity.

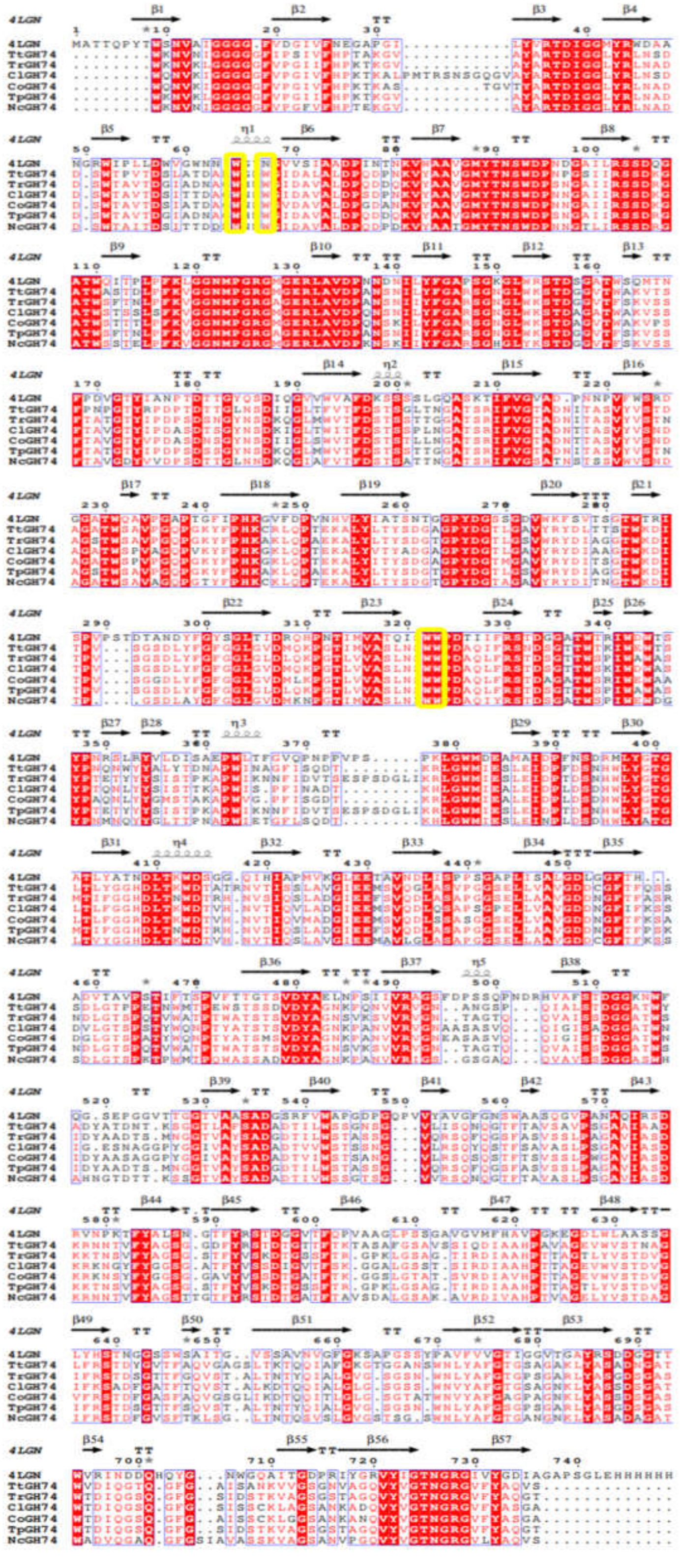

Figure S3. Multiple sequence alignments between *Tt*GH74 and GH74 xyloglucanases from other fungal strains (*Trichoderma reesei* GH74 XP_006966240.1; *Coniochaeta ligniaria* GH74 OIW24422.1; *Coniochaeta sp.* GH74 KAB5566692.1; *Trichoderma parareesei* GH74 OTA06671.1; *Neurospora crassa* GH74 XP_958569.1). Conserved tryptophan residues were indicated by the yellow boxes.

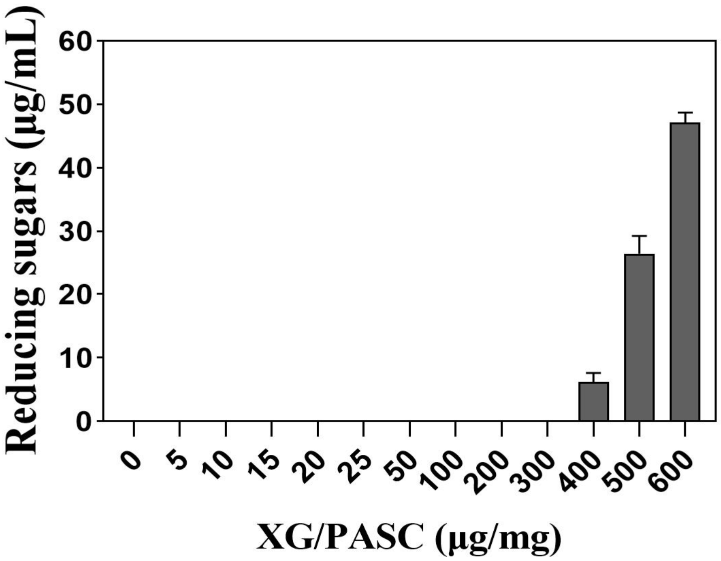

Figure S4. The reducing sugars released from the free XG in supernatant of XG-coated PASC mixture by extensively hydrolysis of overdose *Tt*GH74.The reducing sugar was measured by the DNS method.

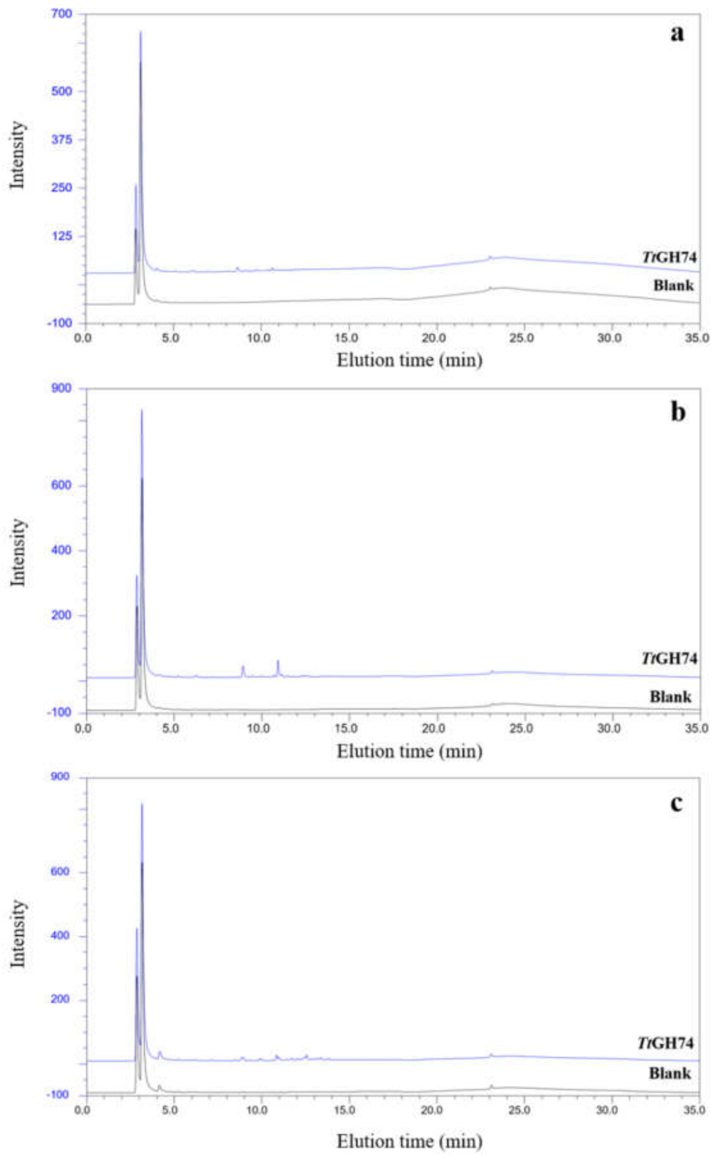

Figure S5. HPAEC-PAD analysis of reaction products generated by *Tt*GH74 from alkali treated residues of sulfuric acid pretreated-corn bran (**a**), DES pretreated-corn bran (**b**) and DES pretreated-apple pomace (**c**). The blank in the HPAEC-PAD represents a control experiment without *Tt*GH74.
